# How organizational justice reduces turnover intention of Korean and non-Korean employees in South Korea: the mediating roles of organizational conflict and job satisfaction

**DOI:** 10.3389/fpsyg.2026.1824345

**Published:** 2026-06-17

**Authors:** Insook Cho, Sung Jun Jo, My Hanh Bui

**Affiliations:** Department of Business Administration, Gachon University, Seongnam, Gyeonggi-do, Republic of Korea

**Keywords:** foreign enterprises, job satisfaction, Korea, organizational conflict, organizational justice, PLS-SEM, turnover intention

## Abstract

**Introduction:**

The high turnover intention has a particularly negative impact on continuous organizational development in South Korea. This study explores the impact of organizational justice on turnover intention, examining the mediating roles of organizational conflict and job satisfaction among employees in foreign enterprises in Korea. It also examines whether these relationships differ by employee nationality.

**Methods:**

The non-probability convenience sampling method was employed to collect and analyze data from 775 employees working in foreign enterprises across the Seoul metropolitan area. Partial least squares structural equation modeling (PLS-SEM) was applied to test the proposed hypotheses. Further, a multi-group analysis (MGA) was conducted to investigate differences in all hypotheses between Korean and non-Korean groups.

**Results:**

The finding revealed that organizational justice positively influenced job satisfaction and negatively affected organizational conflict and turnover intention. Organizational conflict increased turnover intention, whereas job satisfaction reduced it. Both conflict and satisfaction partially mediated the justice-turnover intention link. The results of MGA revealed that most structural relationships were generally similar between Korean and non-Korean employees. However, differences were observed in selected structural relationships, particularly in the relationship between interactional justice and organizational conflict. It indicates the need for culturally responsive HR strategies.

**Discussion:**

This research contributes to the literature on factors influencing turnover intention in foreign enterprises in South Korea, while its limitations underscore the need for future studies to broaden and validate the findings across diverse contexts. From the findings, we offer contributions to talent management in multicultural settings, supporting more effective and inclusive workforce retention strategies.

## 1 Introduction

South Korea has been a member of the World Trade Organization (WTO) since 1995, offering a useful time frame to examine how Korea’s markets and institutional environment have evolved over time. Following its accession to the WTO, the Korean government relaxed market restrictions for foreign investors. Korea offers the best ever for foreign investment. Attracted by its advanced infrastructure, highly educated and skilled workforce, and strategic location, multinational corporations have expanded into Korea across industries.

According to Korea Foreign Company Association (2026), there are currently over 15,000 foreign-invested companies operating in Korea because the business environment is becoming more welcoming to foreign companies. These companies promote Korea’s economic development, like driving industrial development, creating employment, and enhancing global business integration. Korea’s historically homogeneous labor market has become much more diverse as a consequence of this expansion. Demographic challenges, such as low birth rates and an aging population in Korea, have further accelerated the influx of foreigners. The number of foreign workers in Korea exceeded 1.1 million in 2025, out of the 1.69 million foreign residents who are at least 15 years old (Korea Joongang Daily, 2025).

However, even with advances in artificial intelligence (AI) that may substitute some human labor, manpower supply is still projected to lag well behind demand, resulting in the economy is experiencing persistent labor shortages (Choi, 2026). Although the Korean government has introduced numerous efforts to retain talent, high turnover continues to be a serious and ongoing challenge. This undermines organizational performance and workforce stability. Turnover results in significant expenses, up to 213% of the workforce’s annual salary (Lucas, 2012; Boushey and Glynn, 2012). Understanding the psychological processes that influence individuals, leaving the organization continuing to be an ongoing attention in Organizational Psychology and Human Resource Management (HRM).

Among the variables impacting turnover intention, organizational justice is one of the antecedents that has been studied the most (DeConinck and Stilwell, 2004; Li et al., 2022). Justice refers to an individual’s perception that organizational events, actions, and decisions conform to standards of fairness. In organizational contexts, justice perceptions are particularly salient in decision-making processes and outcomes, including promotion and transfer decisions, performance evaluation procedures, fair pay, job placement, and termination. Perceived organizational injustice is a significant cause of employee frustration and job dissatisfaction and is linked to responses involving withdrawal, including absenteeism and turnover. Such reactions may undermine organizational effectiveness and create significant operational challenges, which can have a negative impact on the business (Jung and Hwang, 2019).

Organizational conflict refers to a relational reaction to perceived unfairness. When employees believe that processes or outcomes are unfair, interpersonal conflicts and tensions may rise as a result of frustration and mistrust, all of which heighten organizational conflict (Colquitt et al., 2001; Qin and Zhang, 2022; Huo and Jiang, 2023). As conflict escalates, employees are more likely to feel emotional exhaustion, reduced satisfaction, and stronger intentions to leave (Liu et al., 2015). Unresolved conflict contributes to toxic organizational climates that further erode employee morale and retention (Shaukat et al., 2017).

Employees’ overall evaluation of their workplace is also captured by job satisfaction. Fairness perceptions enhance employees’ sense of value, satisfaction, and motivation, thereby mitigating conflict and enhancing workplace harmony (Nadiri and Tanova, 2010; Bayarçelik and Findikli, 2016; Zhao and Wang, 2023). Whereas injustice diminishes satisfaction and directly intensifies the desire to leave (Kee and Chung, 2021). Investigating both mediators concurrently may thus offer a more comprehensive explanation of the justice-turnover link.

While the justice-turnover link has been extensively studied in Western contexts (Colquitt et al., 2001; Kim and Park, 2017; Pimentel et al., 2025), research focusing on employees in developed Asian economies like Korea remains limited. South Korea’s multicultural workplace context provides an important setting for examining organizational justice and employee outcomes. Prior literature suggests that cultural characteristics such as collectivism (Lee and Ha, 2023), hierarchical workplace structures (Kim et al., 2023), and power distance (Jeon and Seol, 2016), may shape employees’ perceptions of fairness and interpersonal relationships in organizations. However, these cultural factors are discussed in the present study primarily as contextual background rather than as variables directly measured or tested in the research model.

Addressing this gap, the present study investigates how organizational justice influences turnover intention, with organizational conflict and job satisfaction as mediators. Both Korean and non-Korean staff participated in the data collection. This research is particularly timely given the growing multicultural workforce in South Korea. As foreign employees have increased, managing cultural differences and divergent expectations has become a key challenge. By recognizing the factors that lead to turnover intentions, organizations can take proactive steps to reduce actual employee turnover. This study provides academic and practical insights for developing culturally responsive HRM strategies and enhancing high-potential employee retention.

## 2 Theoretical background and hypotheses development

### 2.1 Organizational justice and turnover intention

The concept of organizational justice came from equity theory (Adams, 1965) and has been widely used in organizational behavior research (Chen et al., 2015). Organizational justice is fundamentally grounded in the idea of fairness. It is suggested that employees’ job performance and organizational participation are shaped by the extent to which they perceive their work environment as fair or unfair. Organizational justice reflects employees’ perceptions of fairness in the social and economic exchanges that take place between themselves and their organization (Beugré, 1998). Widely acknowledged as a key driver of trust, satisfaction, and prosocial behavior (Colquitt et al., 2001), it is typically categorized into three dimensions: distributive, procedural, and interactional justice (Greenberg and Lind, 2000). *Distributive justice* pertains to the fairness of outcome allocations—such as pay or rewards—relative to employee contributions (Adams, 1965; Leventhal, 1980). Perceived inequities can lead to dissatisfaction, reduced motivation, and increased organizational conflict (Homans, 1961; Adams, 1965). *Procedural justice* concerns the fairness of the processes guiding decisions. Transparent, consistent, and unbiased procedures foster trust and reinforce legitimacy in organizational practices (Leventhal, 1980; Tyler, 1989; Konovsky and Cropanzano, 1991). *Interactional justice* refers to the quality of interpersonal treatment, particularly respect, dignity, and information sharing (Bies and Shapiro, 1987). Poor interpersonal treatment can undermine satisfaction and elevate turnover intention (Greenberg, 2001). Collectively, these dimensions shape employees’ fairness perceptions, which in turn influence satisfaction, commitment, conflict levels, and retention (Akram et al., 2020; Hoang et al., 2022).

Turnover intention refers to an employee’s conscious consideration of leaving their organization to pursue other opportunities (March and Simon, 1958; Mobley, 1977). It includes thoughts of resignation, job search behaviors, and plans to transition to a different workplace (Yin-Fah et al., 2010; Moon, 2011). While not always resulting in actual departure, turnover intention is a strong predictor of employee exit and poses significant risks to organizational performance and continuity (Steel and Ovalle, 1984; Kim and Lee, 2015). Since it is seen to be the most immediate precursor of real turnover behavior (Daly and Dee, 2006), it has emerged as a central outcome variable in organizational research.

Turnover intention is recognized as one of the most extensively studied forms of withdrawal behavior (Perryer et al., 2010). It serves as a strong predictor of actual turnover and provides a critical basis for early interventions aimed at employee retention. Consequently, managing turnover intention has become a central priority for organizations seeking to maintain workforce stability (Sahi and Mahajan, 2014). According to previous studies, several factors influencing employees’ turnover intention include salary (Berber and Gašić, 2024), recognition (Galanis et al., 2024), limited benefits (Hong et al., 2024), inflexible working hours (Zheng et al., 2025), and restricted career advancement opportunities (Wang et al., 2024). Employees evaluate the fairness of rewards relative to their contributions, aligning with broader research on organizational justice and employee attitudes (Niehoff and Moorman, 1993; Colquitt et al., 2001; Robbins et al., 2015). Prior studies have consistently found a negative relationship between organizational justice and turnover intention (Cohen-Charash and Spector, 2001; Colquitt et al., 2001). So, we propose:

*H1*: Organizational justice is negatively related to turnover intention.

*H1a*: Distributive justice is negatively related to turnover intention.

*H1b*: Procedural justice is negatively related to turnover intention.

*H1c*: Interactional justice is negatively related to turnover intention.

### 2.2 Organizational justice and organizational conflict

Many studies (Mikula and Wenzel, 2000; Goldman et al., 2008; Priesemuth et al., 2013) have indicated that organizational justice has a lot of impact on organizational conflict. When there is injustice, people may concurrently experience contradictory feelings and thoughts about the organization (Pratt, 2000), it may cause the organization to become dysfunctional (Rahim, 1983). In contrast, justice signals acceptance and guarantees the employee valued in the workplace (Kerwin et al., 2015). Key psychological needs can be met via distributive justice, wherein employees are rewarded for their perceived contributions (Adams, 1965). Procedural justice gives individuals a greater sense of control over, and trust in, decision-making processes (Adams, 1965), and interactional justice conveys information about the nature of relationships between group members (Tyler and Lind, 1992). Therefore, the following hypotheses can be formed:

*H2*: Organizational justice is negatively related to organizational conflict.

*H2a*: Distributive justice is negatively related to organizational conflict.

*H2b*: Procedural justice is negatively related to organizational conflict.

*H2c*: Interactional justice is negatively related to organizational conflict.

### 2.3 Organizational justice and job satisfaction

Job satisfaction describes an individual’s overall evaluation of their work and work environment, encompassing both cognitive assessments and emotional responses (Smith et al., 1983; McShane and von Glinow, 2008). It reflects the extent to which work experiences meet personal expectations, with greater alignment linked to higher satisfaction (Dewi and Supartha, 2018). As a key outcome in organizational justice research, job satisfaction captures employees’ perceptions of fairness, workplace conditions, and interpersonal dynamics (Bakhshi et al., 2009).

Social relationships and financial security are critical to satisfaction (Wicaksono et al., 2021), with organizational justice emerging as a significant predictor (Colquitt and Zipay, 2015). Several studies have confirmed a strong positive relationship between perceived justice and job satisfaction (Cohen-Charash and Spector, 2001; Viswesvaran and Ones, 2002; García-Izquierdo et al., 2012). Distributive, procedural, and interactional justice each contribute significantly to satisfaction levels (Bakhshi et al., 2009; Al-Zu’bi, 2010; Tziner et al., 2011; Crow et al., 2012), highlighting the importance of fair organizational practices in fostering favorable employee outcomes. Hence, the following hypotheses were proposed:

*H3*: Organizational justice is positively related to job satisfaction.

*H3a*: Distributive justice is positively related to job satisfaction.

*H3b*: Procedural justice is positively related to job satisfaction.

*H3c*: Interactional justice is positively related to job satisfaction.

### 2.4 Organizational conflict, job satisfaction, and turnover intention

Organizational conflict is a well-established antecedent of turnover intention, primarily due to its detrimental effects on employee stress and job satisfaction (Bedeian and Armenakis, 1981; Milhem et al., 2024). Conflict increases plans to leave the organization and frequently precedes withdrawal behaviors (Gupta and Beehr, 1979). Elevated conflict increases psychological strain and reduces satisfaction, thereby heightening turnover risk (Krausz et al., 1995). Emotional exhaustion contributes to decreased morale and a greater likelihood of departure (Liu et al., 2018). Furthermore, persistent conflict creates toxic work environments that make it harder to retain employees (Browning et al., 2000). These findings highlight the strategic importance of conflict management in improving job satisfaction and reducing turnover intention. Therefore, the following hypothesis can be formed:

*H4*: Organizational conflict is positively related to turnover intention.

Turnover intention is shaped by various factors, such as insufficient compensation, inflexible schedules, and limited career advancement opportunities. However, job satisfaction remains a central determinant of employees’ decisions to remain with or leave an organization (Ghiselli et al., 2001). It reflects both emotional and cognitive evaluations of the work environment, influenced by managerial support, relational dynamics, and alignment between personal and organizational goals (Melnyk, 2006). Prior studies indicate a strong negative relationship between job satisfaction and turnover intention, establishing satisfaction as one of the major predictors of employee retention (Scanlan and Still, 2013; Lee et al., 2017). Thus, the following hypothesis was proposed:

*H5*: Job satisfaction is negatively related to turnover intention.

### 2.5 Organizational conflict as a mediator

Mikula and Wenzel (2000) found that conflict is often linked to feelings of unfairness. That is, when employees believe their company is fair, they tend to show positive outcomes like less conflict, higher levels of commitment, and better job performance (Cropanzano et al., 2007). In contrast, employees’ perceptions of unfairness can result in a string of withdrawal behaviors, with turnover being the most prominent consequence (Conlon et al., 2005). Thus, this leads to the following hypotheses:

*H6*: Organizational conflict mediates the relationship between organizational justice and turnover intention.

*H6a*: Organizational conflict mediates the relationship between distributive justice and turnover intention.

*H6b*: Organizational conflict mediates the relationship between procedural justice and turnover intention.

*H6c*: Organizational conflict mediates the relationship between interactional justice and turnover intention.

### 2.6 Job satisfaction as a mediator

Job satisfaction describes an individual’s overall evaluation of their work and work environment, encompassing both cognitive assessments and emotional responses (Smith et al., 1983; McShane and von Glinow, 2008). It reflects the extent to which work experiences meet personal expectations, with greater alignment linked to higher satisfaction (Dewi and Supartha, 2018). As a key outcome in organizational justice research, job satisfaction captures employees’ perceptions of fairness, workplace conditions, and interpersonal dynamics (Bakhshi et al., 2009).

Previous studies argue that organizational justice influences employees’ workplace attitudes, including organizational commitment and job satisfaction. These positive attitudes contribute to higher organizational success (Martin and Bennett, 1996), as well as play an important role in reducing turnover intention (Zagladi et al., 2015). Robbins and Judge (2009) highlighted that when there is disparity in an organization, it leads to job dissatisfaction and ultimately causes unease and discomfort at work. Employees who feel they are treated unfairly may become alienated within the organization, and if there is no improvement, it in turn increases the intention to quit. Thus, we hypothesize that:

*H7*: Job satisfaction mediates the relationship between organizational justice and turnover intention.

*H7a*: Job satisfaction mediates the relationship between distributive justice and turnover intention.

*H7b*: Job satisfaction mediates the relationship between procedure justice and turnover intention.

*H7c*: Job satisfaction mediates the relationship between interactional justice and turnover intention.

**Figure 1:**
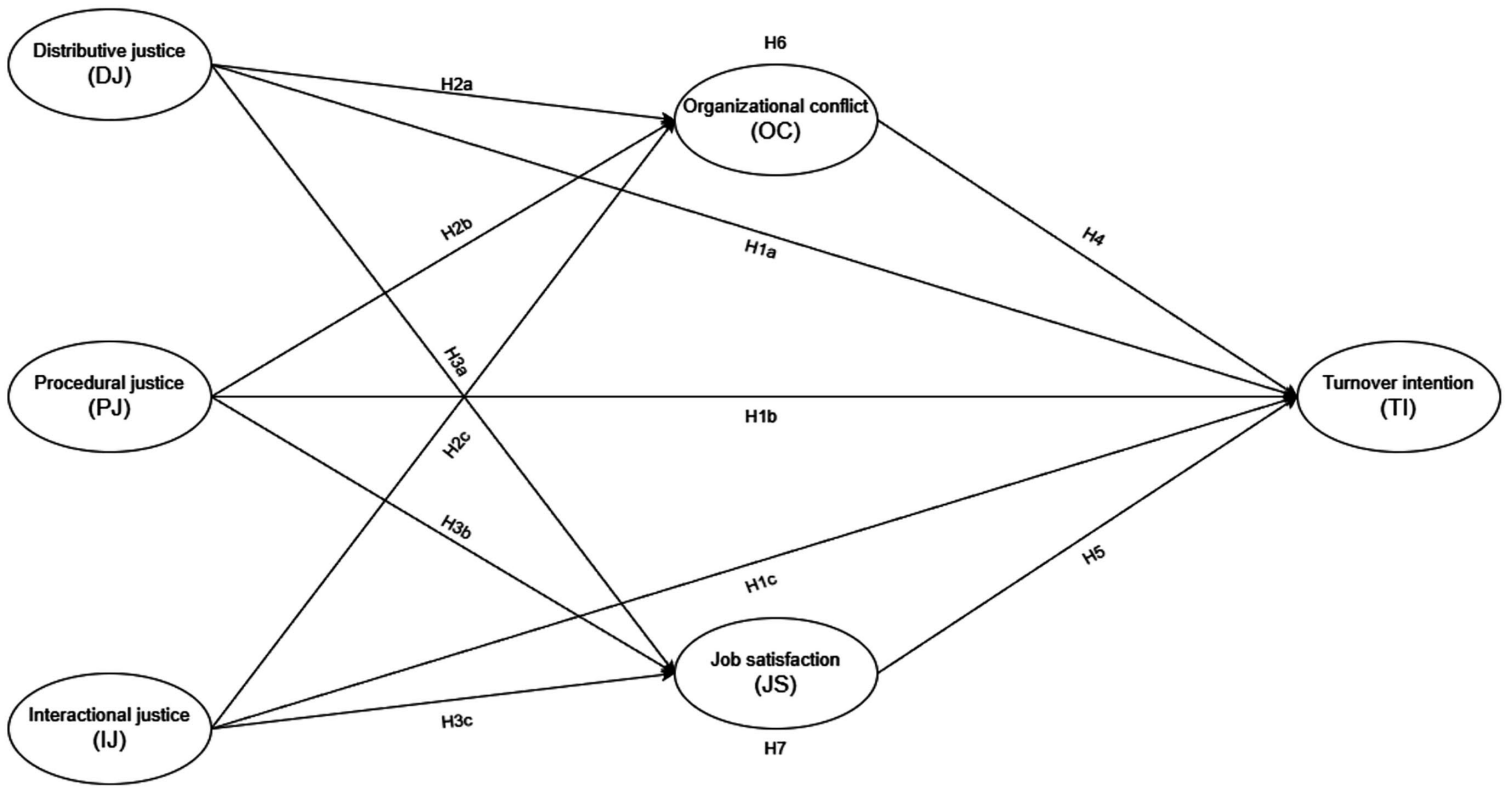
Conceptual framework.

The research framework for this study is shown in the diagram below. Turnover intention is the dependent variable, with organizational justice as the independent variable. Organizational conflict and job satisfaction act as mediating variables, as identified in the literature (Figure 1).

## 3 Methods

### 3.1 Sample and procedure

This study examined the impact of organizational justice (distributive, procedural, and interactional) on turnover intention, with organizational conflict and job satisfaction as mediators. The target population consisted of employees working for multi-national companies in South Korea. Data was collected from July 1 to August 31, 2025 using non-probability convenience sampling method through surveys distributed via Google forms and email. Of the 1,000 surveys distributed, 860 responses were received (response rate: 86%). After excluding 85 incomplete or duplicate responses, 775 valid questionnaires were retained for analysis. Demographic information of survey participants is summarized on Table 1.

**Table 1:** Demographic characteristics of survey participants (*n* = 775).

Profile/category	Option	Option	Percentage
Gender	Male	329	42.5
	Female	446	57.5
Age	20 years old~29 years old	295	38.1
	30 years old~39 years old	232	29.9
	40 years old~49 years old	159	20.5
	More than 50 years old	89	11.5
Nationality	Korean	333	43.0
	Foreigner	442	57.0
Education	High school	123	15.9
	Bachelor	384	49.5
	Master	180	23.2
	Doctoral	88	11.4
Job role in the company	Intern	175	22.6
	Staff	282	36.4
	Team leader	118	15.2
	Manager	123	15.9
	Director	77	9.9
Duration of stay in the company	Less than 1 year	206	26.6
	1 year to 5 years	240	31.0
	6 years to 10 years	120	15.5
	11 years to 15 years	136	17.5
	More than 16 years	73	9.4
Department	Accounting	141	18.2
	Human resource	122	15.7
	Marketing and sales	265	34.2
	Operations and services	144	18.6
	Public relations	67	8.6
	Others	36	4.6
Marital status	Single	305	39.35
	Married	470	60.65
Company size	1 ~ 9	160	20.6
	10 ~ 49	220	28.4
	50 ~ 99	150	19.4
	100 ~ 199	129	16.6
	200 ~ more	116	15.0
Sector of business	Information and communication/IT	194	25.0
	Service	226	29.2
	Finance	140	18.1
	Construction	117	15.1
	Trading	98	12.6

### 3.2 Measurements

All constructions in this study were evaluated using validated scales adopted from previous research. Responses were collected using a five-point Likert scale ranging from 1 (strongly disagree) to 5 (strongly agree). The specific scales are listed below.

*Organizational justice* was measured using Niehoff and Moorman (1993) scale, capturing distributive, procedural, and interactional justice. Distributive justice was evaluated with five items addressing the fairness of outcomes such as pay, workload, and responsibilities. While Moorman et al. (1998) previously reported a Cronbach’s alpha of 0.90, this study obtained a reliability coefficient of 0.847. Procedural justice was measured with six items evaluating fairness in decision-making processes, including accuracy, impartiality, and the opportunity for input. The original scale showed a reliability of 0.90 (Niehoff and Moorman, 1993), the current study reported an alpha of 0.895. Interactional justice was measured through nine items examining the respectfulness and clarity of managerial communications. The original reliability was 0.90 (Niehoff and Moorman, 1993), and the present study achieved a Cronbach’s alpha of 0.937.

*Organizational conflict* was measured using a combination of Jehn (1995) scale and adaptations from Rahim and Magner (1995), Jehn et al. (2008), Hjertø and Kuvaas (2009). The eight items captured task-related conflict and relationship conflict among coworkers. Reliability analysis yielded Cronbach’s alpha coefficients of 0.92 for the relationship conflict scale and 0.87 for the task conflict scale. For this study, the overall reliability coefficient alpha was 0.883.

*Job satisfaction* was measured using a four-item scale developed by Lucas et al. (1990), which assessed compensation, work conditions, and overall job contentment. Cronbach’s alpha was 0.838 in this study, compared to 0.83 in the original scale.

*Turnover intention*, defined as an employee’s psychological tendency to leave their organization (Mobley, 1977), was measured using four items from Kalbers and Fogarty (1995) and three items from Viator (2001). The reliability of the scale computed using Cronbach’s alpha was 0.926.

### 3.3 Data analysis methods

SPSS version 26 was used for preliminary data screening, descriptive statistics, and Harman’s single-factor test to assess the potential presence of common method bias in the dataset (Hair et al., 2010). In addition, AMOS version 24 was employed to conduct the Common Latent Factor (CLF) procedure for further assessing potential common method bias. Structural equation modeling (SEM) was employed to analyze the proposed research model. SEM includes two main types: covariance-based SEM (CB-SEM) for theory testing and partial least squares SEM (PLS-SEM) for exploratory research focusing on maximizing explained variance (Hair et al., 2014). Given the exploratory nature and complexity of the model, PLS-SEM was selected, using SmartPLS 4.0 software (Chin, 1998; Ringle et al., 2005; Tenenhaus et al., 2005; Esposito Vinzi et al., 2010). The analysis followed a two-step approach: evaluation of the measurement model and the structural model.

### 3.4 Common method bias assessment

The current study used self-reported data collected through a survey questionnaire, which meant potential for a common method bias (CMB) problem as each participant responded to a survey (Podsakoff et al., 2003). Therefore, the current study adopted Harman’s Single factor test as it is the most widely used test to assess CMB. In the study, the variance explained was calculated at 40.708%, less than the 50% criterion suggested by Harman (1976). Statistical results showed that CMB was not a serious issue in the current study.

To further assess common method bias, the Common Latent Factor (CLF) technique was conducted using AMOS. A common latent factor was added to the measurement model and connected to all observed indicators with equal loading constraints. Following the recommendation of Lowry and Gaskin (2014), the standardized factor loadings before and after including the CLF were compared. The differences between the standardized loadings were all below the recommended threshold of 0.20, indicating that common method bias was not a serious concern in this study.

## 4 Results

### 4.1 Descriptive statistics

Table 2 presents the descriptive statistics of the study constructs, including the mean and standard deviation values. The results indicate relatively high levels of distributive justice, procedural justice, interactional justice, and job satisfaction among respondents, whereas turnover intention showed the lowest mean value. Organizational conflict demonstrated a moderate level.

**Table 2:** Descriptive statistics.

Variable	Item	Mean	SD
DJ	5	4.09	0.68
PJ	6	4.10	0.74
IJ	9	4.01	0.72
OC	8	2.33	0.65
JS	4	4.36	0.74
TI	7	1.64	0.66

**Table 3:** Result of the VIF coefficient.

Construct	DJ	IJ	JS	OC	PJ	TI
DJ			1.431	1.431		1.717
IJ			1.303	1.303		1.511
JS						2.133
OC						1.953
PJ			1.503	1.503		1.860
TI						

### 4.2 Variance inflation factor

The variance inflation factor (VIF) was used to assess multicollinearity among the predictor constructs in the structural model. According to Hair et al. (2011), VIF values below 5 are acceptable, while Kock (2015) recommends a stricter threshold of 3.3. All VIF values in this study were below 3, as determined using the SmartPLS 4 algorithm, confirming that there are no multicollinearity problems among the predictor latent variables (Hair et al., 2017a; Hair et al., 2017b) (Table 3).

### 4.3 Reliability and convergent validity

When evaluating internal consistency, this study examined both Cronbach’s alpha and composite reliability (CR), in line with the recommendations of (Hair et al., 2017a; Hair et al., 2017b). The analysis results in Table 4 show that both Cronbach’s alpha and CR are greater than the threshold value (*α* ≥ 0.60; CR ≥ 0.70) for all values, support adequate convergent validity (Cronbach, 1951; Hair et al., 2017a; Hair et al., 2017b). The average variance extracted (AVE) was calculated to evaluate convergent validity. It can be seen from Table 4 that the AVE of each variable is greater than 0.5, thereby indicating the convergent validity of the measurement model (Fornell and Larcker, 1981; Henseler et al., 2009). Additionally, all item loadings were above 0.70, demonstrating strong indicator reliability (Chin, 2010).

**Table 4:** Overall evaluation index of measurement model.

Construct	Cronbach’s alpha	CR	AVE	Factor loadings
DJ	0.847	0.891	0.620	[0.767–0.813]
PJ	0.895	0.919	0.655	[0.794–0.824]
IJ	0.937	0.947	0.666	[0.794–0.833]
OC	0.883	0.907	0.549	[0.721–0.756]
JS	0.838	0.891	0.673	[0.806–0.830]
TI	0.926	0.940	0.692	[0.819–0.841]

### 4.4 Discriminant validity

Discriminant validity was assessed using the Fornell-Larcker criterion and the Heterotrait-Monotrait (HTMT) ratio (Henseler et al., 2015). Following Fornell and Larcker (1981), the square root of the AVE for each construct exceeded its correlations with other constructs. As shown in Table 5, the diagonal AVE values were consistently greater than the off-diagonal correlations, confirming satisfactory discriminant validity.

**Table 5:** Fornell-Larcker criterion.

Construct	1	2	3	4	5	6
DJ	**0.787**					
IJ	0.394	**0.816**				
JS	0.545	0.516	**0.820**			
OC	−0.574	−0.508	−0.589	**0.741**		
PJ	0.517	0.442	0.628	−0.536	**0.810**	
TI	−0.613	−0.653	−0.670	0.622	−0.575	**0.832**

Bold values on the diagonal represent the square roots of the Average Variance Extracted (AVE). Discriminant validity is established when the square root of the AVE for each construct is greater than its correlations with other constructs in the corresponding row and column.

HTMT represents the ratio of average correlations between indicators across constructs (heterotrait-heteromethod) to those within the same construct (monotrait-heteromethod). Lower HTMT values indicate stronger discriminant validity. Discriminant validity is established when HTMT values are below 0.85 (Henseler et al., 2015; Kline, 2023) or 0.90 (Gold et al., 2001; Teo et al., 2008). Additionally, HTMT values significantly below one further confirms discriminant validity (Memon et al., 2018). Based on these criteria, discriminant validity was deemed satisfactory (Table 6).

**Table 6:** The results of Heterotrait-Monotrait ratio (HTMT) test.

Construct	1	2	3	4	5	6
DJ						
IJ	0.440					
JS	0.646	0.580				
OC	0.663	0.557	0.685			
PJ	0.593	0.482	0.724	0.602		
TI	0.691	0.699	0.760	0.686	0.630	

### 4.5 Model fit

Table 7 shows the structural model fit indices. The standardized root mean square residual (SRMR) was 0.037, which is below the suggested threshold of 0.08, indicating satisfactory model fit (Hair et al., 2022). In addition, the normed fit index (NFI) value was 0.928, exceeding the recommended threshold of 0.90 and further supporting acceptable model fit (Hu and Bentler, 1998). The d_ULS and d_G values also indicated an acceptable model fit. Overall, these results suggest that the proposed model demonstrates adequate fit for the study (Sarstedt and Cheah, 2019).

**Table 7:** Model fit.

Fit index	Saturated model	Estimated model
SRMR	0.034	0.037
d_ULS	0.922	1.079
d_G	0.301	0.308
Chi-square	1336.981	1354.926
NFI	0.929	0.928

### 4.6 Evaluation of structural (inner) model

Following the successful assessment of the measurement model, the structural model was evaluated using effect size (ƒ^2^), coefficient of determination (R^2^), predictive relevance (Q^2^), as recommended by Hair et al. (2014). The ƒ^2^, calculated as [ƒ^2^ = (R^2^included − R^2^excluded)/(1 − R^2^included)], reflects the strength of predictor variables. Following Cohen (2013), ƒ^2^ values of 0.35, 0.15, and 0.02 represent large, medium, and small effects, respectively. Higher ƒ^2^ values indicate stronger predictive relationships between constructs.

The R^2^ values for job satisfaction (0.507) and organizational conflict (0.462) reflect moderate and weak-to-moderate predictive power, respectively, while the R^2^ for turnover intention (0.651) indicates moderate-to-substantial predictive power (Hair et al., 2017a; Hair et al., 2017b). These findings suggest that the structural model demonstrates satisfactory explanatory capability.

**Table 8:** ƒ^2^, R^2^, Q^2^ results.

Path	ƒ^2^	*R* ^2^	Q^2^
DJ → OC	0.155		
DJ → JS	0.086		
DJ → TI	0.088		
IJ → OC	0.101		
IJ → JS	0.094		
IJ → TI	0.210		
PJ → OC	0.071		
PJ → JS	0.208		
PJ → TI	0.012		
OC → TI	0.026		
JS → TI	0.077		
OC		0.462	0.458
JS		0.507	0.504
TI		0.651	0.605

The Q^2^ was assessed using the blindfolding procedure. A Q^2^ value greater than zero indicates predictive relevance (Garson, 2016), with thresholds of 0.02, 0.15, and 0.35 representing weak, moderate, and strong predictive power, respectively. In this study, Q^2^ values for job satisfaction (0.504), organizational conflict (0.458), and turnover intention (0.605) exceeded zero (Hair et al., 2017a; Hair et al., 2017b), confirming that the model has strong predictive relevance for all endogenous constructs (Table 8).

### 4.7 Hypotheses testing

**Figure 2:**
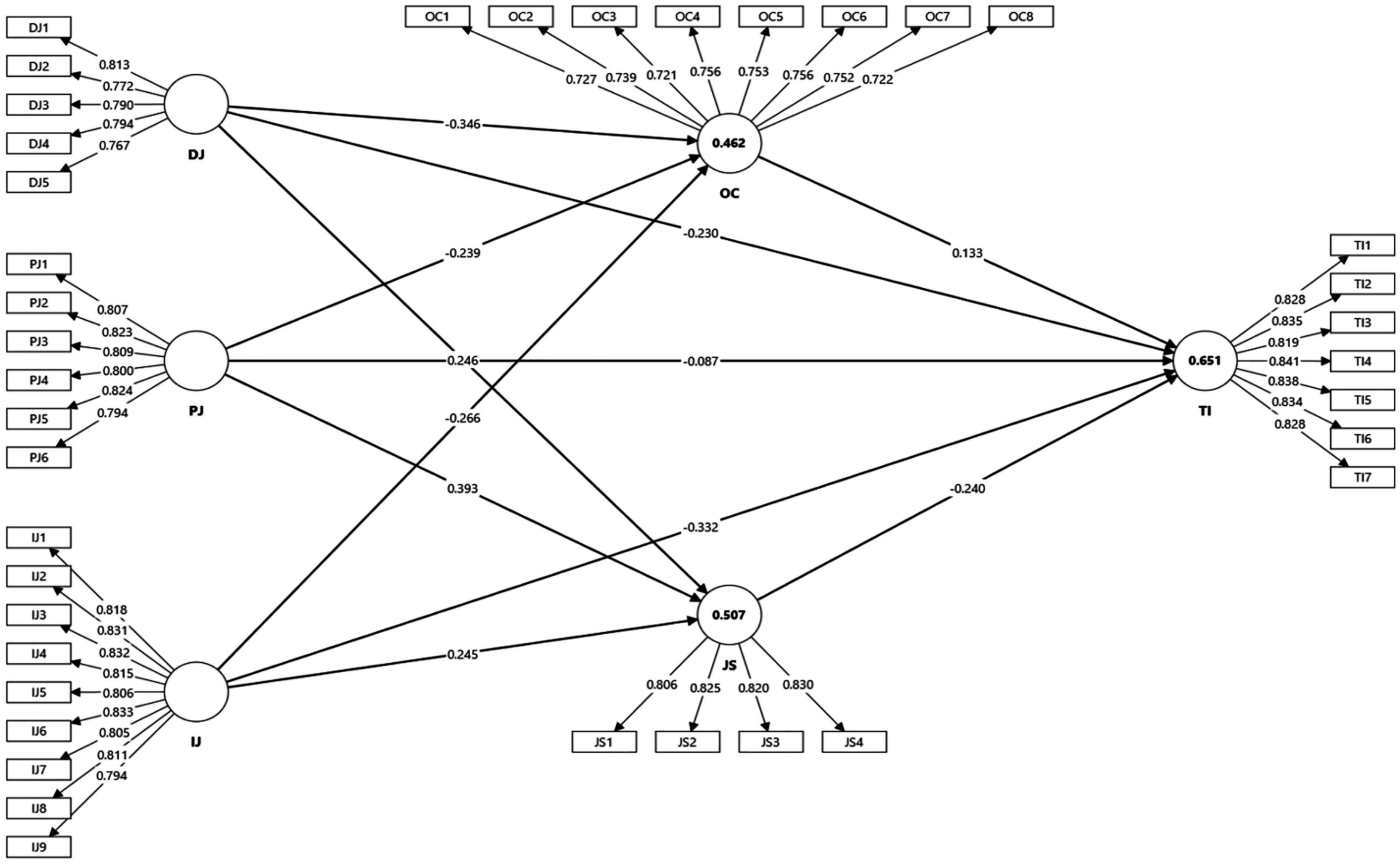
Results of structural tests.

As shown in Table 9 and Figure 2, distributive justice, procedural justice, and interactional justice were significantly negatively related to organizational conflict and turnover intention, while positively related to job satisfaction. The results support H1a–H3c. In addition, organizational conflict was significantly positively associated with turnover intention (*β* = 0.133, *t* = 4.418, *p* < 0.001), whereas job satisfaction was significantly negatively associated with turnover intention (*β* = −0.240, *t* = 7.757, *p* < 0.001), confirming H4 and H5.

**Table 9:** Path coefficient results.

Path	Original sample (O)	Sample mean (M)	Standard deviation (STDEV)	T statistics (|O/STDEV|)	*P*-values	Supported
H1a: DJ - > TI	−0.230	−0.228	0.027	8.486	0.000	Yes
H1b: PJ - > TI	−0.087	−0.086	0.028	3.073	0.002	Yes
H1c: IJ - > TI	−0.332	−0.332	0.024	14.073	0.000	Yes
H2a: DJ - > OC	−0.346	−0.345	0.030	11.426	0.000	Yes
H2b: PJ - > OC	−0.239	−0.240	0.030	7.939	0.000	Yes
H2c: IJ - > OC	−0.266	−0.265	0.029	9.254	0.000	Yes
H3a: DJ - > JS	0.246	0.246	0.032	7.634	0.000	Yes
H3b: PJ - > JS	0.393	0.392	0.032	12.113	0.000	Yes
H3c: IJ - > JS	0.245	0.245	0.029	8.467	0.000	Yes
H4: OC - > TI	0.133	0.132	0.030	4.418	0.000	Yes
H5: JS - > TI	−0.240	−0.241	0.031	7.757	0.000	Yes

### 4.8 Mediation analysis

Table 10 presents the results of mediation analysis. The analysis was conducted by bootstrapping procedures based on 5,000 subsamples to assess the significance of indirect effects (Zhao et al., 2010). The findings reveal that both organizational conflict and job satisfaction partially mediate the relationships between distributive, procedural, and interactional justice and turnover intention. These results imply empirical support for the proposed theoretical framework and underscore the importance of both conflict management and employee satisfaction in understanding how organizational justice influences turnover intention.

**Table 10:** Results of mediation analysis.

Path	Original sample (O)	Sample mean (M)	Standard deviation (STDEV)	T statistics (|O/STDEV|)	*P*-values	Supported
H6a: DJ- > OC - > TI	−0.046	−0.046	0.011	4.182	0.000	Yes
H6b: PJ - > OC - > TI	−0.032	−0.032	0.008	3.857	0.000	Yes
H6c: IJ - > OC - > TI	−0.035	−0.035	0.009	4.117	0.000	Yes
H7a: DJ - > JS - > TI	−0.059	−0.060	0.012	4.755	0.000	Yes
H7b: PJ - > JS - > TI	−0.094	−0.094	0.014	6.537	0.000	Yes
H7c: IJ - > JS - > TI	−0.059	−0.059	0.011	5.530	0.000	Yes

### 4.9 Measurement invariance

Before performing the multi-group analysis (MGA), the measurement invariance of composite models (MICOM) was conducted to examine invariance in the groups under consideration (Henseler et al., 2016). The sample was divided into Korean (*n* = 333) and non-Korean (*n* = 442) groups. The MICOM approach includes three steps: (1) configural invariance, (2) compositional invariance, (3) equality of means and variances. If configural invariance (step 1) and compositional invariance (step 2) are established, partial measurement invariance is confirmed, and the path coefficients can be compared with the MGA. If partial measurement invariance is verified and the composites exhibit equal means and variances across the groups, the full measurement invariance is verified. Table 11 reports the result of the MICOM assessment. According to Table 11, configural and compositional invariance were established for all constructs, implying partial measurement invariance. Therefore, the subsequent multigroup analysis (MGA) was considered valid and meaningful.

**Table 11:** Results of invariance measurement testing using permutation.

Construct	Compositional invariance correlation = 1			Partial measurement invariance established	Equality of measures		Equality of variances		Full measurement invariance established
	Configure invariance	C = 1	95%C1		Difference	Confidence interval 95%	Difference	Confidence interval 95%	
DJ	Yes	1.000	0.999	Yes	−0.009	[−0.152, 0.138]	0.010	[−0.318, 0.304]	Full
IJ	Yes	1.000	1.000	Yes	−0.012	[−0.138, 0.134]	−0.036	[−0.320, 0.287]	Full
JS	Yes	1.000	0.999	Yes	−0.015	[−0.138, 0.141]	−0.003	[−0.422, 0.347]	Full
OC	Yes	0.999	0.999	Yes	0.049	[−0.150, 0.153]	−0.022	[−0.327, 0.288]	Full
PJ	Yes	1.000	0.999	Yes	0.063	[−0.145, 0.129]	−0.026	[−0.268, 0.244]	Full
TI	Yes	1.000	1.000	Yes	0.018	[−0.138, 0.145]	0.021	[−0.610, 0.532]	Full

### 4.10 Multigroup analysis

To assess whether participant nationality (Korean vs. non-Korean) influenced the proposed research relationships, a PLS-SEM multigroup analysis (PLS-MGA) was performed. In PLS-MGA, a difference between groups is considered significant when the *p*-value is above 0.95 or below 0.05 (Henseler et al., 2016). Overall, the analysis showed that most structural relationships did not significantly differ across the two nationalities (*p* > 0.05), suggesting that the effects of distributive, procedural, and interactional justice on organizational conflict, job satisfaction, and turnover intention were largely consistent for both groups. However, two paths demonstrated statistically significant differences between Korean and non-Korean employees. First, the path from interactional justice to organizational conflict differed significantly (*p* < 0.05). Specifically, the negative effect of interactional justice on organizational conflict was stronger among Korean employees (*β* = −0.338) than among non-Korean employees (*β* = −0.212). This finding suggests that Korean employees may be more sensitive to interpersonal fairness and communication quality within the organization. Second, the relationship between procedural justice and turnover intention approached significance (*p* = 0.054), although it did not reach the conventional threshold of *p* < 0.05. The negative effect of procedural justice on turnover intention was stronger for Korean employees (*β* = −0.151) than for non-Korean employees (*β* = −0.040). This result suggests that fair organizational procedures may play a relatively more important role in reducing turnover intention among Korean employees. However, this finding should be interpreted cautiously because the difference between groups was not statistically significant at the conventional level. Overall, the findings indicate that nationality was associated with differences in selected structural relationships rather than representing a formal moderating effect (Table 12).

## 5 Discussion

This study examined the impact of organizational justice (distributive, procedural, interactional) on turnover intention. Furthermore, the study explored the mediating effects of both organizational conflict and job satisfaction on this relationship. The findings confirm the significant role of fairness perceptions in shaping employee attitudes and behavioral intentions within multicultural work environments.

Research findings are in line with previous research that organizational justice has a negative effect on the intention to leave a job (Mengstie, 2020; Pimentel et al., 2025). Distributive justice was found to be one of the strongest predictors in the model among the three dimensions of organizational justice. Specifically, distributive justice significantly reduced organizational conflict and turnover intention while positively affecting job satisfaction. This finding suggests that employees in multinational companies place considerable importance on the fairness of organizational outcomes such as salary, workload distribution, and promotion opportunities. In multicultural workplaces like Korea, comparisons between Korean and non-Korean employees may become more noticeable, making employees more sensitive to unequal treatment or perceived inequities. Results from our study reaffirm the view that fairness perceptions contribute to lower and more positive attitudes toward the workplace (Nadiri and Tanova, 2010).

The findings suggest that procedural justice significantly reduces turnover intention and organizational conflict, while increasing job satisfaction. These findings suggest that employees value consistent, transparent, unbiased decision-making processes. Fair decision-making processes may strengthen employees’ trust in the workplace, enhance their perceptions of organizational support, and lower their dissatisfaction and intention to leave. In the context of multinational companies in Korea, hierarchical organizational structures and centralized decision-making practices may also influence how employees perceive procedural justice. Employees may be relatively familiar with formal organizational hierarchies, which could partially explain why procedural justice showed a weaker influence on turnover intention compared to distributive justice.

Interactional justice also showed a strong negative relationship with organizational conflict and turnover intention. This finding highlights the importance of respectful communication, interpersonal treatment, and managerial behavior in multinational organizational environments. Employees who perceive respectful treatment from leaders and coworkers are less likely to experience organizational conflict or develop intentions to leave the organization.

The mediation analysis further revealed that organizational conflict and job satisfaction played important mediating roles in the relationship between organizational justice and turnover intention. Employees who perceive greater fairness are less likely to experience organizational conflict and are more likely to report stronger job satisfaction, which subsequently lowers their intention to leave the organization. In multinational organizational settings, where employees may experience difficulties with cultural adaptation, challenges with workplace adjustment and communication barriers, job satisfaction becomes especially important for preserving employee retention organizational commitment.

The result of the MGA indicates that nationality differences appeared only in selected structural relationships rather than across all paths in the model. This suggests that Korean and non-Korean employees have similar perceptions of several aspects of organizational justice while differing in specific areas related to organizational experience and expectation. Although this study did not directly measure cultural factors such as hierarchy, collectivism, and communication norms, these contextual characteristics may partially help to explain why certain relationships differed between the two groups. Employees’ perceptions of fairness, interpersonal treatment, and organizational conflict in multinational environments may be influenced by hierarchical workplace structures and indirect communication techniques in South Korean organizational settings.

Overall, the findings emphasize the importance of considering both organizational and contextual factors when analyzing employee attitudes and turnover intention in multicultural workplace settings.

### 5.1 Theoretical and practical implications

#### 5.1.1 Theoretical implications for researchers

This study contributes to organizational justice literature in several ways.

First, the study extends previous research by simultaneously examining the effects of distributive justice, procedural justice, and interactional justice on organizational conflict, job satisfaction, and turnover intention within multinational companies in South Korea. While prior studies have largely focused on general organizational settings, this study provides evidence from a multicultural organizational context in which employees from different national and cultural backgrounds work together.

Second, this study contributes to the literature by demonstrating the mediating roles of organizational conflict and job satisfaction in explaining how organizational justice influences turnover intention. The findings suggest that fairness perceptions affect employee retention not only directly, but also indirectly through employees’ emotional experiences and workplace relationship dynamics. In particular, the stronger mediating role of job satisfaction indicates that employees’ psychological and attitudinal responses are important mechanisms linking fairness perceptions to turnover intention.

Third, by examining Korean and non-Korean employees within the same organizational context, this study arguably offers new perspectives on organizational justice in multicultural workplace environments.

#### 5.1.2 Practical implications for companies in the Asian context

The findings provide actionable guidance for organizations aiming to improve employee retention by enhancing perceptions of organizational justice.

First, equitable reward distribution is essential. Compensation, benefits, and recognition should align with employee contributions and capabilities to reduce dissatisfaction and strengthen retention (Figueiredo et al., 2025). A well-structured rewards system—including performance-based incentives, career development, and flexible work options—can significantly boost satisfaction (Bryant and Allen, 2013; Mamun and Hasan, 2017). Since distributive justice emerged as the strongest predictor of turnover intention, managers should carefully monitor employees’ perceptions of fairness regarding compensation, promotion opportunities, workload distribution, and organizational rewards.

**Table 12:** Multigroup analysis results.

Path	Korean	Non-Korean	Diff.	PLS MGA (p)
DJ - > TI	−0.174	−0.271	0.097	0.081
PJ - > TI	−0.151	−0.04	−0.110	0.054
IJ - > TI	−0.343	−0.324	−0.019	0.693
DJ - > OC	−0.335	−0.353	0.017	0.775
PJ - > OC	−0.209	−0.267	0.058	0.332
IJ - > OC	−0.338	−0.212	−0.125	0.028*
DJ - > JS	0.25	0.244	0.006	0.928
PJ - > JS	0.362	0.417	−0.055	0.386
IJ - > JS	0.289	0.211	0.078	0.182
OC - > TI	0.136	0.132	0.004	0.951
JS - > TI	−0.227	−0.249	0.022	0.731

Second, organizations should promote fair and transparent organizational procedures. Consistent and unbiased decision-making processes may help improve employee trust, increase job satisfaction, and reduce turnover intention. Managers should clearly communicate organizational policies and provide employees with opportunities to express their opinions during decision-making processes.

Third, the strong influence of interactional justice suggests that respectful communication and interpersonal treatment are particularly important in multicultural workplaces. Managers should therefore develop communication practices that emphasize mutual respect, fairness, and cultural sensitivity. Training programs related to leadership communication, conflict management, and intercultural understanding may help reduce organizational conflict and improve workplace relationships.

The findings also indicate that organizational conflict and job satisfaction are important mechanisms linking fairness perceptions to turnover intention. Therefore, organizations should actively reduce workplace conflict while simultaneously promoting employee satisfaction through fair treatment, transparent communication, and supportive organizational practices.

Finally, multinational companies should recognize that employees from different cultural backgrounds may respond differently to organizational experiences. Therefore, organizations should develop culturally sensitive human resource management practices that consider communication styles, organizational expectations, and workplace relationship norms among both Korean and non-Korean employees.

Importantly, the results highlight the necessity of HR strategies that support effective communication and inclusive organizational environments. Employees from diverse cultural backgrounds may understand fairness and workplace interactions differently. Company efforts like clear communication channels, consistent organizational policies, and equal opportunities can improve organizational trust, help employees adapt, and strengthen long-term organizational stability.

### 5.2 Limitations and suggestions for future research

This study still has some limitations. First, reliance on self-reported data raises the risk of common method bias, particularly in collectivist cultures where social desirability may influence responses. Future studies should adopt multi-source and longitudinal designs incorporating supervisor ratings and HR data to fortify the research design and improve the reliability of the results. Second, this study focused exclusively on employees in multinational enterprises located in the Seoul metropolitan area, which may limit the generalizability of findings. Future research should include diverse industries, larger firms, and rural regions. Additional multigroup analyses by nationality, visa status, or region may uncover nuanced patterns among domestic and foreign workers. Third, justice perceptions may differ in Asian contexts due to strong cultural norms like hierarchy and collectivism, and the sample used in this study is not representative of Western cultural settings. Future research should examine these contexts to assess the cross-cultural validity and strengthen external generalizability.

## 6 Conclusion

In conclusion, this study examined the relationships among organizational justice, organizational conflict, job satisfaction, and turnover intention, as well as the mediating roles of organizational conflict and job satisfaction, using data collected from 775 respondents in South Korea, including both Korean and non-Korean employees. The findings revealed significant direct and indirect (mediated) effects, providing strong empirical support for all proposed hypotheses. These results highlight the crucial influence of organizational justice in shaping employees’ satisfaction, reducing conflict, and ultimately lowering turnover intention. This research’s findings and implications may give HR experts and researchers insights into addressing the continual talented employee shortage by controlling the turnover intention-causing forces in multinational enterprises.

## Data Availability

The raw data supporting the conclusions of this article will be made available by the authors, without undue reservation.
